# Gigantol ameliorates CCl_4_-induced liver injury via preventing activation of JNK/cPLA2/12-LOX inflammatory pathway

**DOI:** 10.1038/s41598-020-79400-0

**Published:** 2020-12-17

**Authors:** Yaru Xue, Qiangqiang Deng, Qingli Zhang, Zhenghua Ma, Binfan Chen, Xiaolu Yu, Huige Peng, Sheng Yao, Jia Liu, Yang Ye, Guoyu Pan

**Affiliations:** 1 Shanghai Institute of Materia Medica, Chinese Academy of Sciences, Shanghai, 201203 China; 2 University of Chinese Academy of Sciences, Beijing, 100049 China; 3 Institutional Technology Service Center, Shanghai Institute of Materia Medica, Chinese Academy of Sciences, Shanghai, 201203 China; 4 State Key Laboratory of Drug Research and Natural Products Chemistry Department Shanghai, Institute of Materia Medica, Chinese Academy of Sciences, Shanghai, 201203 China; 5 SIMM-CUHK Joint Research Laboratory for Promoting Globalization of Traditional Chinese Medicines, Shanghai, 201203 China; 6 School of Life Science and Technology, Shanghai Tech University, Shanghai, 201203 China

**Keywords:** Drug discovery, Molecular biology, Plant sciences, Diseases, Medical research, Pathogenesis

## Abstract

Arachidonic acid (AA) signaling pathway is an important constituent of inflammatory processes. In our previous study, it was found that dihydro-stilbene gigantol relieved hepatic inflammation in mice with CCl_4_-induced acute liver injury. This study aimed to investigate the involvement of arachidonate metabolic cascade in this process. Our results showed CCl_4_ activated AA metabolism with the evidence of cPLA2 phosphorylation, which was dependent on the MAPK/JNK activation. Pretreatment with JNK inhibitor SU3327 or gigantol abolished the cPLA2 activation, along with the attenuation of liver damage. Besides, gigantol markedly decreased immune cells activation. Metabolomic analysis revealed that gigantol universally reversed the upregulation of major AA metabolites in injured mouse livers induced by CCl_4_, especially 12-hydroxyeicosatetraenoic acid (12-HETE). Gigantol also decreased the mRNA and protein expression of platelet-, and leukocyte-type 12-lipoxxygenase (LOX) in the liver. Furthermore, pan-LOX inhibitor nordihydroguaiaretic acid (NDGA) and specific 12-LOX inhibitors baicalein and ML351 attenuated the liver injury to the same extent as gigantol. Overall, our study elucidated a comprehensive profile of AA metabolites during hepatic inflammation caused by CCl_4_, highlighting the role of 12-LOX-12-HETE pathway in this process. And gigantol alleviated liver inflammation partly through inhibiting the JNK/cPLA2/12-LOX pathway.

## Introduction

Inflammatory response is the common pathology of various liver diseases, despite the different etiologies. Arachidonic acid (AA) metabolism provides an interesting link between lipid metabolism and inflammation. Under normal physiological conditions, esterified AA is presented in position 2 of membrane phospholipids. When the cell membrane is disturbed by external stresses, the esterified AA can be deesterified and liberated from the membrane by cytosolic phospholipase A2 (cPLA2) with the aid of calcium ions. The free AA in cells can be metabolized by non-enzymatic pathway and three enzymatic pathways, including lipoxygenases (LOX), cytochrome P450 enzymes (CYP450) and cyclooxygenase (COX) pathways. These metabolic pathways will transform AA into a series of oxidized metabolites collectively described as eicosanoids^1^. In the liver, eicosanoids can be produced by Kupffer cells, endothelial cells, recruited leukocytes, as well as hepatocytes^2^. The pro-inflammatory eicosanoids like leukotrienes (LTs) and hydroxyeicosatetraenoic acids (HETEs) can aggravate liver inflammation by further activating macrophages and recruiting neutrophils. And it has been reported that blocking these metabolites by inhibitors or genetic depletion can alleviate the liver diseases^3–5^. However, according to our current knowledge, the metabolomic profile of AA metabolites and the related signaling pathways involved in liver injuries have not been thoroughly investigated.

CCl_4_ is a typical hepatotoxic chemical that is widely used to investigate the pathological mechanisms of liver injury and evaluate the hepatoprotective effect of novel compounds^6^. Several signaling pathways, like MAPK/JNK pathway^7^, and Akt/NF-κB pathway^8^, have been known to account for CCl_4_-induced hepatotoxicity. But the role of arachidonate metabolic cascade in this process and its crosstalk with the above pathways are obscure. Previous research discovered that the LTs derived from 5-LOX catalysis contributed to the pathogenesis of CCl_4_-induced liver injury^9^. But the role of other eicosanoids was not deeply studied. It’s in great need to figure out the beneath mechanisms of AA metabolism in liver injuries.

This study aimed to further investigate the role of different arachidonate metabolic cascades in the pathogenesis of CCl_4_-induced hepatotoxicity and to explore potential intervention strategies. Our previous study showed that dihydro-stilbene gigantol had great anti-inflammatory effects both in vitro and in vivo. For example, it alleviated the CCl_4_-induced liver inflammation in mice. Serum levels of TNF-α, IL-6, the hepatic mRNA expression of pro-inflammatory cytokines (TNF-α, IL-6 and IL-1β) and chemokines (MCP-1 and ICAM-1) were all reversed by gigantol^10^. Here, we used it as a probe drug to assess the potential intervention approach by targeting the AA metabolic pathway.

We clarified the comprehensive AA metabolites profile in the CCl_4_-induced liver injury model by using UPLC/MS/MS-based targeted metabolomics for the first time. Our results highlighted the role of 12-LOX-12-HETE axis in the process of hepatic inflammation. Gigantol modulated the phenotype of immune cells and reversed AA metabolism, possibly through inhibiting JNK/cPLA2/12-LOX pathway. Moreover, specific LOX inhibitors were employed to validate the effects of potential pathways. The elucidation of the AA metabolites profile and key signaling pathways during liver inflammation will provide clues for developing new therapeutics to treat acute liver injury.

## Results

### CCl_4_-induced JNK activation led to the activation of cPLA2

**Figure 1:**
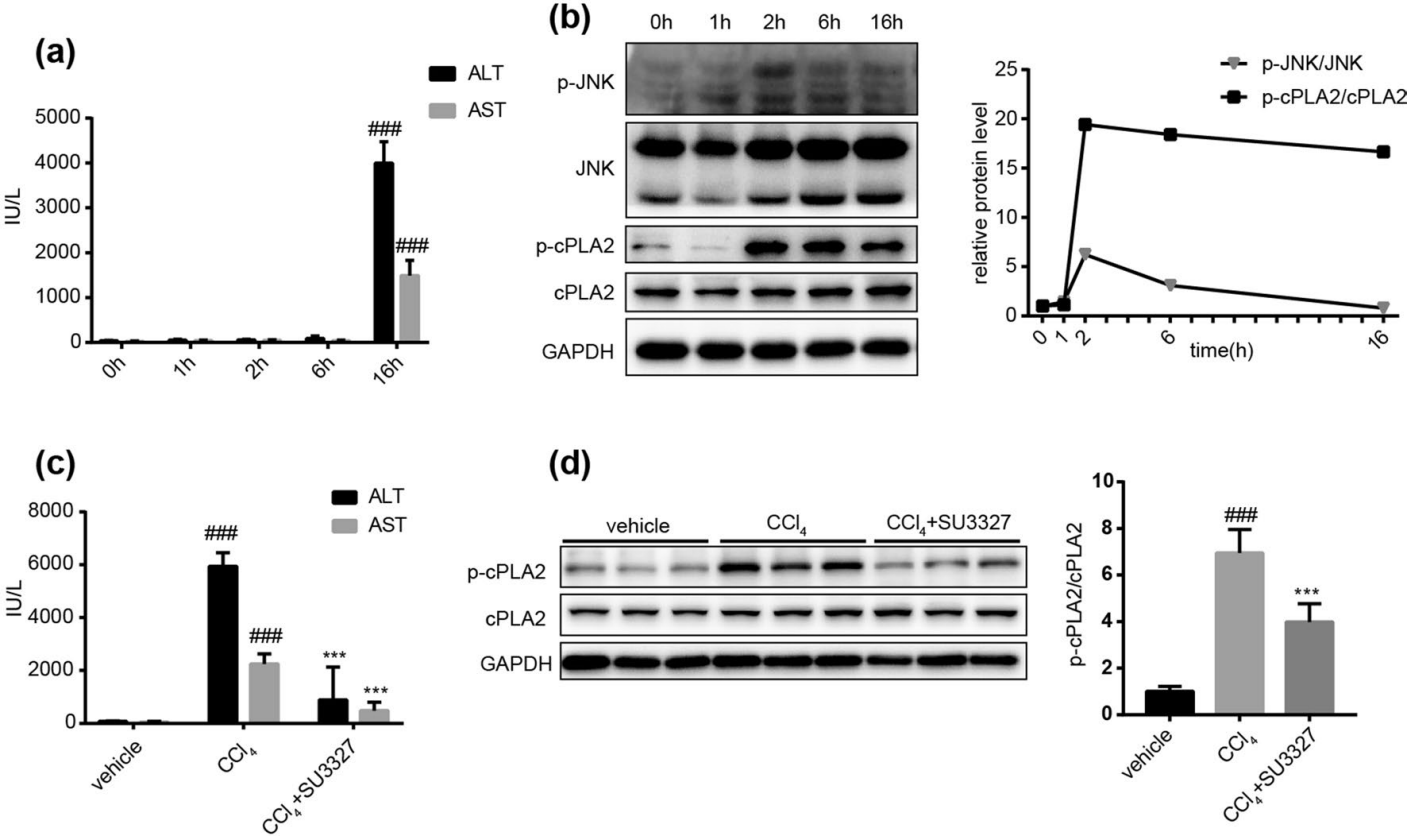
The time dependent changes of JNK and cPLA2 activation and their role in CCl_4_-induced liver injury. (**a**) Time dependent changes in mouse serum ALT and AST levels post CCl_4_ injection. Data are represented as mean ± SD, n = 6. ^###^*P* < 0.001 versus other time points. (**b**) The protein expression of p-JNK, JNK, p-cPLA2, cPLA2 in mouse livers at different time points post CCl_4_ injection. The ratio of p-JNK/JNK and p-cPLA2/cPLA2 is quantified on the right. (**c**) Serum ALT and AST levels in mice with or without a single dose of SU3327 (10 mg kg^−1^, *ip*) 1 h before CCl_4_ administration. (**d**) The protein expression of p-cPLA2 and cPLA2 in mice with or without SU3327 pretreatment at 16 h post CCl_4_ injection. The ratio of p-cPLA2/cPLA2 is quantified on the right. n = 3 western blot experiments. n = 6 mice in each group. The data are shown as the mean ± SD. ^###^*P* < 0.001 versus the vehicle group; ***P < 0.001 versus the CCl_4_ group. Full-length blots are presented in Supplementary Figures S3 and S4.

The time-dependent assays showed that serum ALT and AST remained unchanged within the first 6 h after CCl_4_ administration, but markedly increased at 16 h post CCl_4_ injection (Fig. 1a). However, JNK phosphorylation was rapidly activated upon CCl_4_ stimulation before the pathogenic process. The ratio of p-JNK/JNK reached the maximum at 2 h post CCl_4_ injection and dropped down to the basal level at 16 h point (Fig. 1b). In order to investigate whether CCl_4_ stimulated the release of AA from cell membrane, we also detected the protein expression of p-cPLA2 and cPLA2 at different time points. The results showed that the phosphorylation of cPLA2 started from 2 h post CCl_4_ injection and lasted until 16 h point, as well as the ratio of p-cPLA2/cPLA2 (Fig. 1b). Pretreatment of JNK inhibitor SU3327 could significantly downregulate the serum ALT and AST levels at 16 h post CCl_4_ injection (Fig. 1c). Concurrently, the activation of cPLA2 was blocked by JNK inhibitor SU3327 (Fig. 1d). Overall, these results indicated that CCl_4_ stimulated the activation of cPLA2 in a JNK-dependent way, which contributed to the pathogenesis of liver injury.

### Gigantol significantly attenuated the liver injury and hepatic inflammation

**Figure 2:**
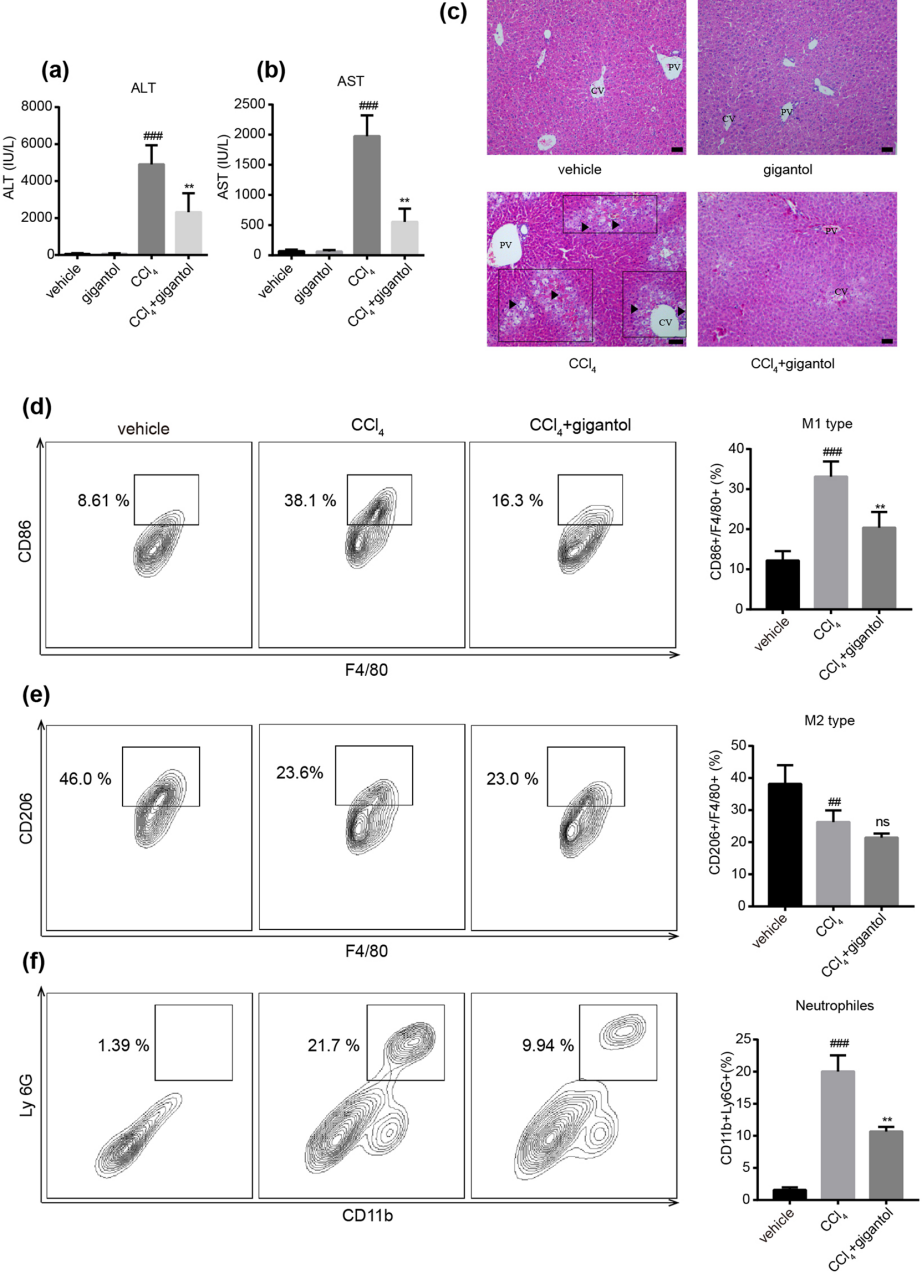
Oral administration of gigantol alleviated CCl_4_-induced liver inflammation in mice. (**a**) Serum ALT and (**b**) AST levels in mice with or without gigantol pretreatment (40 mg kg^−1^ d^−1^, *po*, for 7 days) were measured 16 h post CCl_4_ injection. (**c**) Representative histological sections of liver tissues were stained with H&E. Symbols: black arrow-centrilobular necrosis; rectangle-tumefaction and steatosis; solid triangle-inflammatory infiltration. CV, centrilobular veins; PV, portal veins. Representative flow charts showing (**d**) the percent of M1 type macrophages (CD86+F4/80+) gated by F4/80; (**e**) the percent of M2 type macrophages (CD206+F4/80+) gated by F4/80+ and (**f**) the percent of neutrophils (CD11b+Ly6G+) cells in the liver. The double positive cells are marked by rectangle and the quantitative results are represented on the right. The images were created with FlowJo 7.6 software (https://www.flowjo.com/). Thequantitative results are shown as the mean ± SD, n = 6 mice for each group. ^###^*P* < 0.001, ^##^*P* < 0.01 versus the vehicle group; **P < 0.01 versus the CCl_4_ group.

The protective effect of gigantol on CCl_4_-induced liver injury were demonstrated by the serum aminotransferase levels and the pathological changes. Our results indicated that oral administration of gigantol for 7 days did not affect the function of normal livers, but could reverse the elevated ALT and AST levels 16 h after CCl_4_ injection (Fig. 2a,b). At the same time, the changes in liver histology were attenuated by gigantol (Fig. 2c).

The anti-inflammatory effects were validated by the phenotypes of macrophages and infiltration of neutrophils. The FACS analysis indicated that CCl_4_ significantly increased the percent of M1-type macrophages (marked by CD86, gated by F4/80) (Fig. 2d), decreased the percent of M2-type macrophages (marked by CD206, gated by F4/80) (Fig. 2e) and increased the amount of neutrophils (double-marked by CD11b and Ly-6G) (Fig. 2f) in the livers. The pretreatment of gigantol markedly decreased the M1 polarization of macrophages (Fig. 2d) and inhibited the infiltration of neutrophils into the liver (Fig. 2f). However, gigantol did not impact the percent of M2-type macrophages (Fig. 2e). Collectively, the above results proved that gigantol protected the liver against CCl_4_-induced injury by exerting potent anti-inflammatory effects.

### Gigantol inhibited the activation of JNK and cPLA2

**Figure 3:**
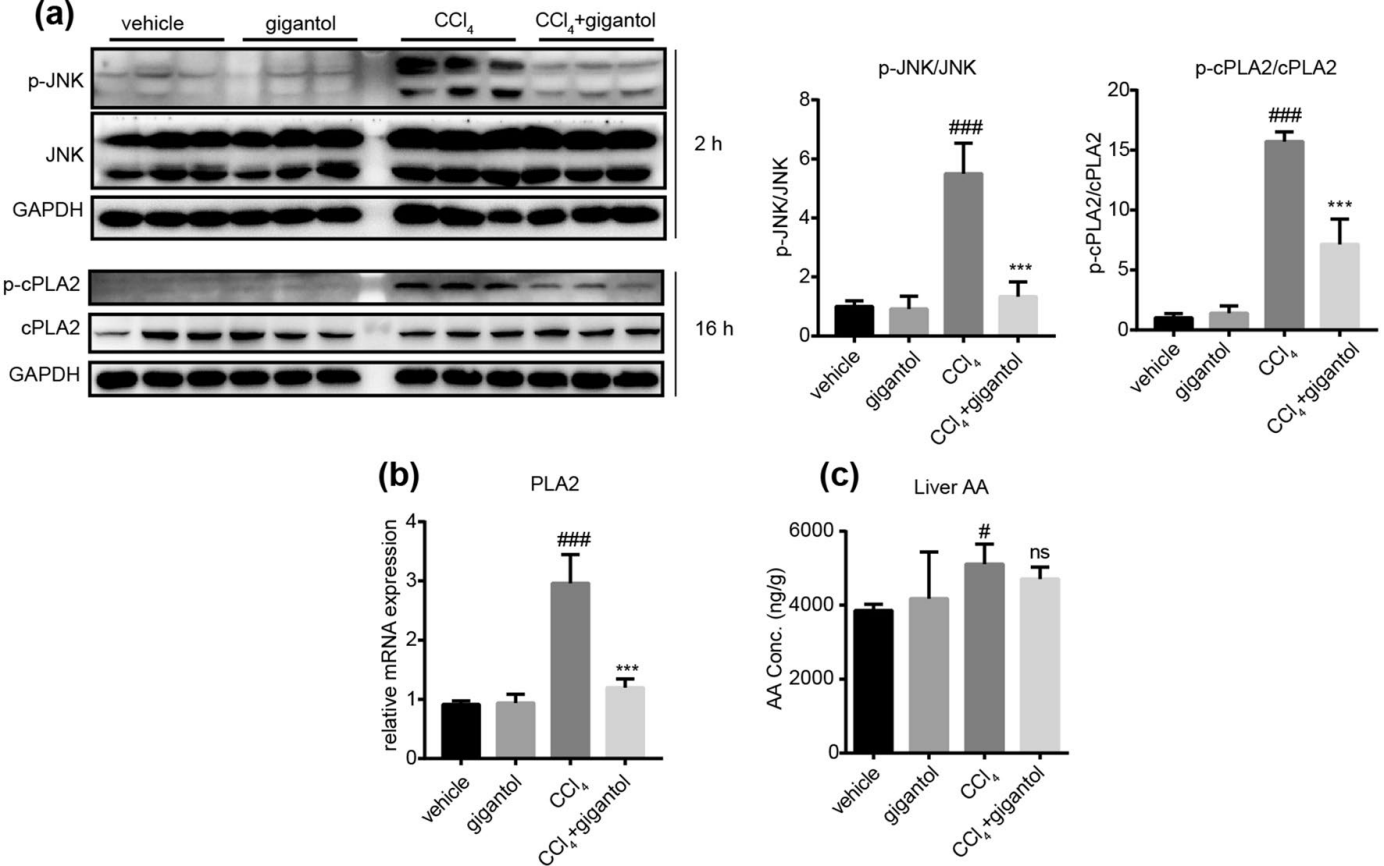
Effect of gigantol on the JNK and cPLA2 activation in livers induced by CCl_4_. (**a**) The protein expression of p-JNK, JNK, p-cPLA2 and cPLA2 in mice with or without gigantol pretreatment (40 mg kg^−1^ d^−1^, *po*, for 7 days) at 2 h or 16 h post CCl_4_ injection. The representative western blot bands are shown, n = 3 western experiments. The ratio of p-JNK/JNK and p-cPLA2/cPLA2 is quantified on the right. (**b**) The mRNA expression of PLA2 in the mouse livers were measured by real-time PCR. (**c**) The concentration of AA in the livers was measured by UPLC/MS/MS. The above results are shown as the mean ± SD, n = 6 mice in each group. ^###^*P* < 0.001, ^##^*P* < 0.05 versus the vehicle group; ***P < 0.001 versus the CCl_4_ group; ns means non-significance. Full-length blots are presented in Supplementary Figure S5.

We further investigated the effect of gigantol on JNK and cPLA2 activation at 2 h and 16 h post CCl_4_ injection respectively. The western blot results showed that gigantol inhibited the protein expression of p-JNK and p-cPLA2 without changing the total protein expression of JNK and cPLA2 (Fig. 3a). The ratio of p-JNK/JNK and p-cPLA2/cPLA2 were quantitatively represented and found to be markedly inhibited by gigantol (Fig. 3a). Real-time PCR was conducted to detect the mRNA expression of PLA2 in the liver, which was inhibited by gigantol (Fig. 3b). Furthermore, the UPLC/MS/MS analysis indicated that the total concentration of AA in mouse liver was mildly increased by CCl_4_ and not significantly influenced by gigantol (Fig. 3c). In summary, gigantol inhibited the activation of JNK and cPLA2, indicating its effect on the AA metabolic pathways.

### Gigantol regulated arachidonate metabolic cascades, especially the 12-LOX-12-HETE pathway induced by CCl_4_

**Figure 4:**
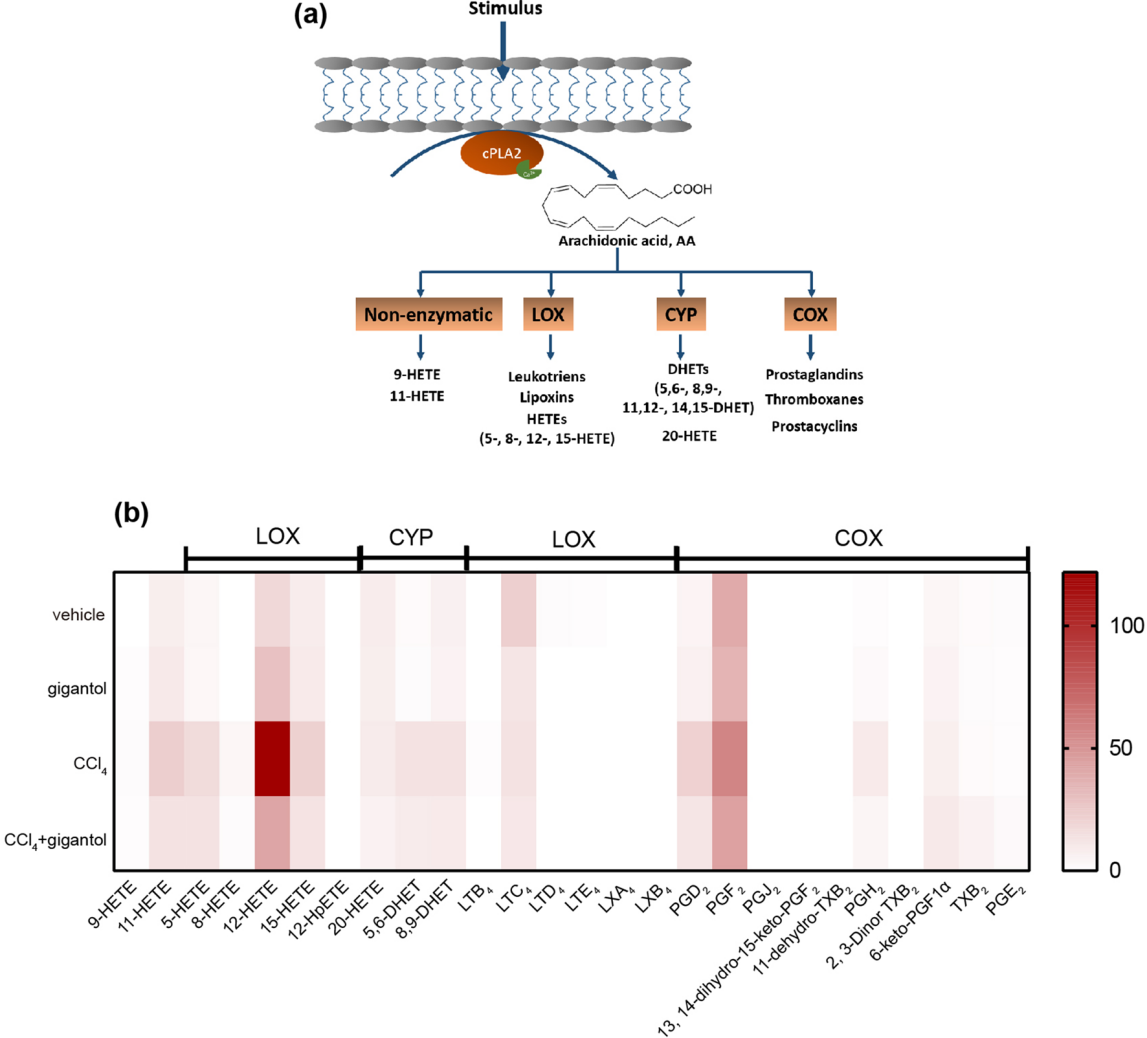
Gigantol reversed the activated AA metabolism induced by CCl_4_. (**a**) The metabolic pathway of AA. Esterified AA located in the cell membrane is catalyzed by cPLA2 with the aid of calcium. The free AA is then metabolized by non-enzymatic pathway and three metabolic pathways. The schematic figure was created by using Adobe Illustrator CC 2019 (https://www.adobe.com/products/illustrator.html) (**b**) Mouse livers with or without gigantol pretreatment (40 mg kg^−1^ d^−1^, *po*, for 7 days) were collected at 16 h post CCl_4_ injection and used for the metabolomics analysis with UPLC/MS/MS. The heat map showing the contents of major AA metabolites catalyzed by non-enzymatic pathway (the left two columns), lipoxygenases (LOX), cytochrome P450 enzymes (CYP) and cyclooxygenase (COX). The heat map was generated by using GraphPad Prism 7.0 software (https://www.graphpad.com/). The above results are shown as the mean, n = 6 mice for each group.

In order to investigate the role of CCl_4_ and gigantol in the AA metabolism, liver samples intoxicated by CCl_4_ with or without gigantol pretreatment were subjected into the UPLC/MS/MS-based targeted metabolomics. The concentration of major AA metabolites in the liver catalyzed by the non-enzymatic pathway, the LOX, CYP450 and COX pathways (Fig. 4a), were quantitatively measured. As shown in the heat map (Fig. 4b), the major AA metabolites were universally elevated by CCl_4_ and reversed by gigantol. Gigantol alone did not affect their levels significantly. Fold changes of AA metabolites concentration between the CCl_4_ group and vehicle group or CCl_4_ + gigantol group were summarized (Supplementary Table S1). CCl_4_ markedly increased the liver concentrations of 11-HETE (non-enzymatic pathway), 5-HETE, 8-HETE, 12-HETE, 15-HETE (LOX pathways), dihydroxyeicosatrienoic acids (5,6-DHET and 8,9-DHET; CYP450 pathways) andprostaglandins (PGD_2_ and PGH_2_; COX2 pathway). Among all the tested AA metabolites, the level of 12-HETE catalyzed by platelet-, and leukocyte-type 12-LOX was most upregulated by CCl_4_ (fold change = 6.67) and reversed by gigantol (fold change = 2.85) (Fig. 4b, Supplementary Table S1). Generally, the lipidomic analysis revealed that the hepatoprotective effect of gigantol was closely relevant to the regulation of arachidonate metabolic cascades, especially the 12-LOX-12-HETE pathway.

### Gigantol inhibited the gene and protein expression of 12-LOX enzymes

**Figure 5:**
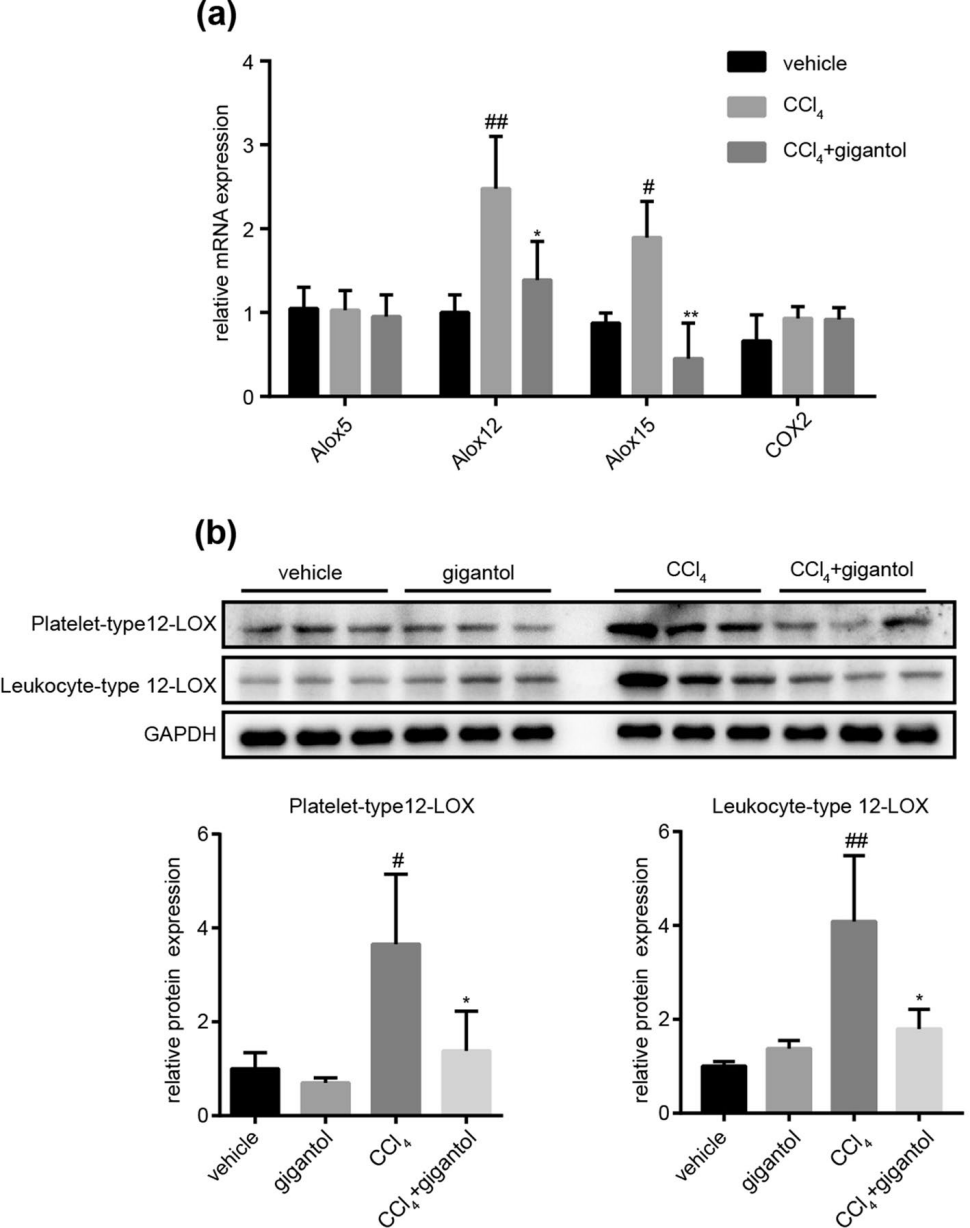
Gigantol significantly inhibited the 12-LOX pathway. (**a**) The relative mRNA expression of Alox5, Alox12, Alox15 and COX2 in the mouse livers from the vehicle, CCl_4_ and (CCl_4_+ gigantol) groups. (**b**) The protein expression of platelet-, and leukocyte-type 12-LOX in the mouse livers at 16 h post CCl_4_ injection. The representative western blot bands are shown, n = 3 western blot experiments. The quantitative results are represented as the mean ± SD, n = 6 mice in each group. ^##^*P* < 0.01, ^#^*P* < 0.05 versus the vehicle group; **P < 0.01, *P < 0.05 versus the CCl_4_ group. Full-length blots are presented in Supplementary Figure S6.

Furthermore, we verified the effect of gigantol on the mRNA and protein expression of 12-LOX in mouse livers intoxicated by CCl_4_. At the same time, we detected the mRNA expression of Alox5 and COX2 as negative control. The RT-PCR results showed that CCl_4_ increased the mRNA expression of Alox12 and Alox15, which were markedly inhibited by gigantol (Fig. 5a). And the mRNA expression of Alox5 and COX2 were not significantly influenced by gigantol (Fig. 5a), which corresponded well with the metabolomic results (Fig. 4b, Supplementary Table S1). Also, the protein expression of platelet-, and leukocyte-type 12-LOX were elevated by CCl_4_ and inhibited by gigantol (Fig. 5b). These results implied that the hepatoprotective effect of gigantol may largely attribute to its inhibitory effect on 12-LOX expression in the liver.

### The pan-LOX inhibitor NDGA exerted similar effect with gigantol on liver injury and inflammatory responses

**Figure 6:**
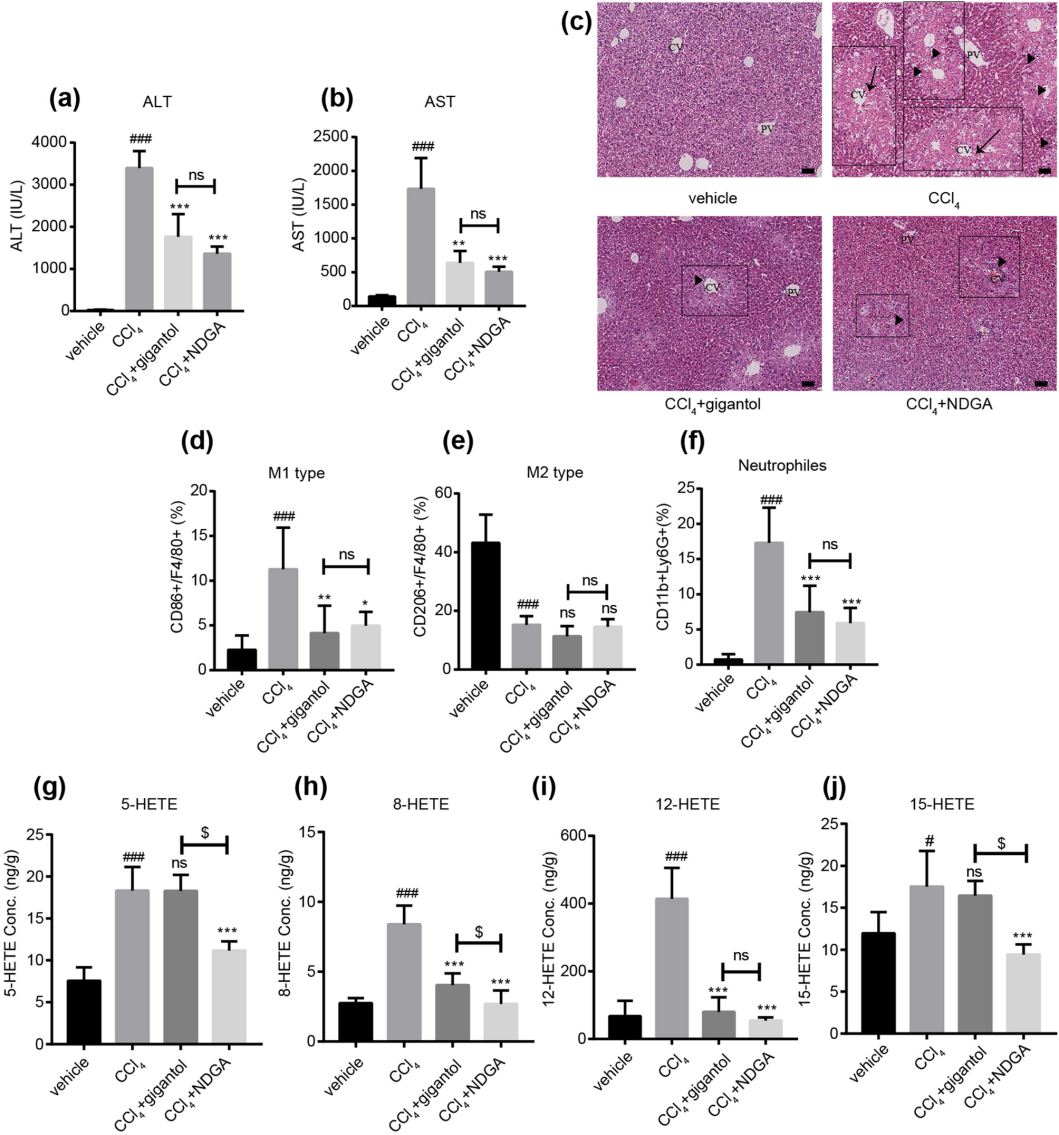
LOX inhibitor NDGA alleviated liver inflammation to the same extent as gigantol. (**a**) Serum ALT and (**b**) AST levels in mice with or without gigantol or NDGA (40 mg kg^−1^ d^−1^, *po*, for 7 days) pretreatment were measured 16 h post CCl_4_ injection. (**c**) Representative histological sections of liver tissues were stained with H&E. Symbols: black arrow-centrilobular necrosis; rectangle-tumefaction and steatosis; solid triangle-inflammatory infiltration. CV-centrilobular veins; PV-portal veins. The percent of (**d**) M1-type macrophages (CD86+F4/80+), (**e**) M2-type macrophages (CD206+F4/80+) and (**f**) neutrophils (CD11b+Ly6G+) were detected by FACS. The concentration of (**g**) 5-HETE, (**h**) 8-HETE, (**i**) 12-HETE, and (**j**) 15-HETE were quantified with UPLC/MS/MS. The above results are shown as the mean ± SD, n = 6 in each group. ^###^*P* < 0.001, ^#^*P* < 0.05 versus the vehicle group; ***P < 0.001, **P < 0.01, *P < 0.05 versus the CCl_4_ group; ^$$$^*P* < 0.001, ^$^*P* < 0.05 (CCl_4_+ gigantol) versus (CCl_4_+ NDGA); ns means non-significance; ns means non-significance.

Pan-LOX inhibitor NDGA^11^ was employed to validate the role of LOX pathways in the pathogenesis of CCl_4_ and the protective mechanism of gigantol. NDGA was administrated in the same way as gigantol (40 mg kg^−1^ day^−1^, 7 days, *po*). Oral administration of gigantol and NDGA downregulated the serum levels of ALT and AST (Fig. 6a,b), as well as the pathological morphology in the livers (Fig. 6c). And the protective effects of NDGA and gigantol were at a comparable level. Moreover, we tested the effect of NDGA on the phenotypes of macrophages and the infiltration of neutrophils. The FACS analysis revealed that NDGA could also inhibit the M1 polarization of macrophages and the infiltration of neutrophils, but had no effect on the M2-type macrophages (Fig. 6d–f). The above effects of NDGA had no significant difference between gigantol.

Next, we compared the inhibitory effects of gigantol and NDGA on the AA metabolites catalyzed by lipoxygenases. The results indicated that NDGA, a pan-LOX inhibitor, reduced the formation of 5-HETE, 8-HETE, 12-HETE and 15-HETE in the mouse liver (Fig. 6g–j). And gigantol only inhibited the formation of 8-HETE and 12-HETE (Fig. 6h,i), which was consistent with the lipidomic results (Fig. 4b, Supplementary Table S1). Particularly, no significant difference was found in the 12-HETE concentrations between the gigantol and NDGA pretreatment groups.

The above results further highlighted the importance of 12-HETE in the pathogenesis of CCl_4_-induced liver injury and the protective mechanism of gigantol.

### Specific 12-LOX inhibitors ameliorated hepatotoxicity caused by CCl_4_

**Figure 7:**
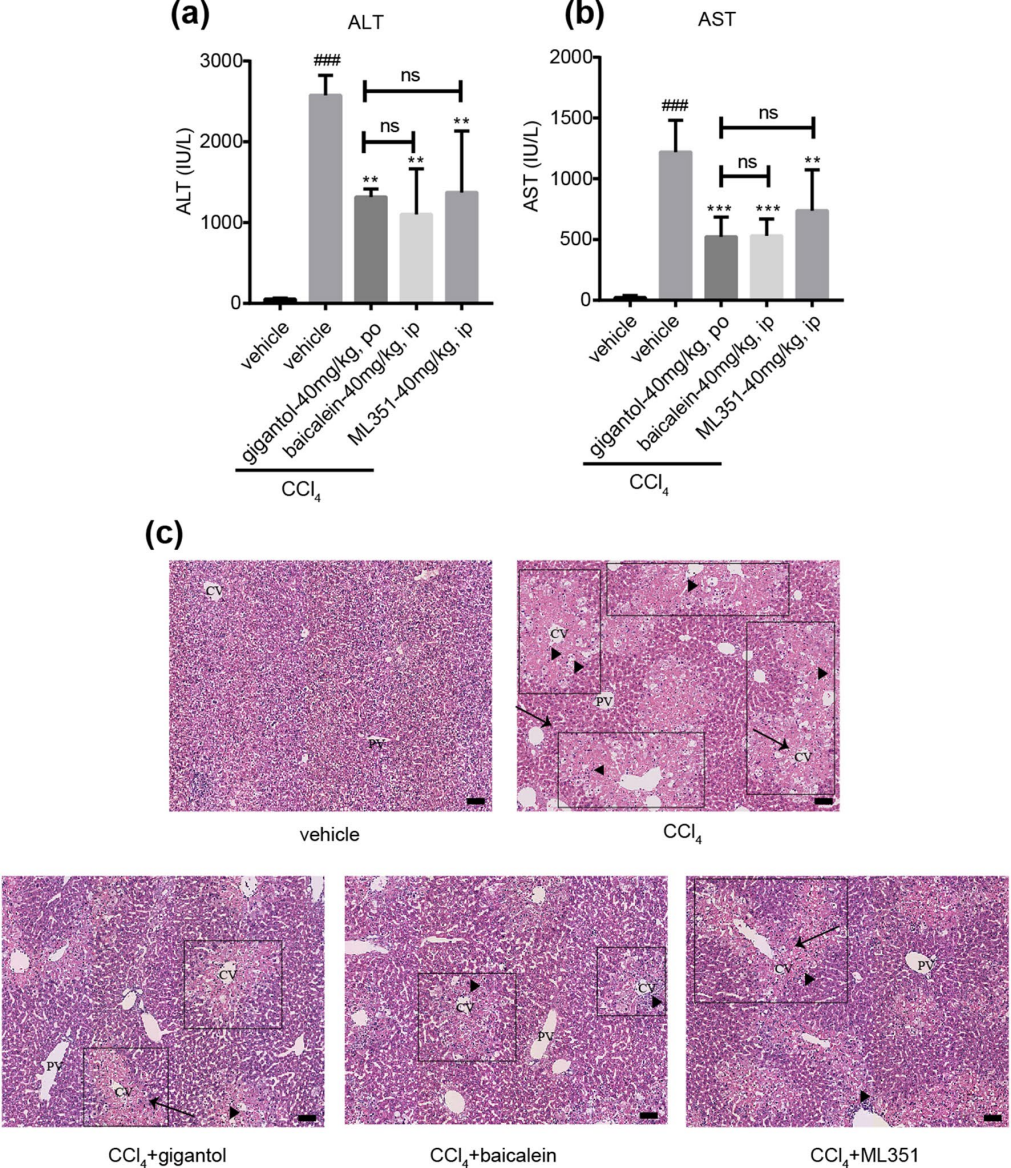
12-LOX inhibitors ameliorates CCl_4_-induced liver injury to the same extent as gigantol. (**a**) Serum ALT and (**b**) AST levels in mice with or without gigantol (40 mg kg^−1^ d^−1^, *po*, for 7 days), baicalein (40 mg kg^−1^ d^−1^, *ip*, for 3 days) and ML351 (40 mg kg^−1^, *ip*, 3 times within 24 h) pretreatment were measured at 16 h post CCl_4_ injection. (**c**) Representative histological sections of liver tissues were stained with H&E. Symbols: black arrow-centrilobular necrosis; rectangle-tumefaction and steatosis; solid triangle-inflammatory infiltration. CV-centrilobular veins; PV-portal veins. The results are shown as the mean ± SD, n = 6 in each group. ^###^*P* < 0.001 versus the vehicle group; ***P < 0.001, **P < 0.01 versus the CCl_4_ group; ns means non-significance.

At last, the platelet-type 12-LOX inhibitor baicalein^12^ and leukocyte-type 12-LOX inhibitor ML351^13^ were employed to verify the role of 12-HETE. The results showed that both baicalein and ML351 pretreatment improved the serum ALT, AST levels and the hepatic pathological changes (Fig. 7a–c). And the differences between gigantol group and baicalein group or ML351 group were not significant. The results suggested specific inhibition of 12-LOX could also alleviate the liver injury induced by CCl_4_, further validating the importance of 12-LOX-12-HETE pathway in the pathogenesis of CCl_4_-induced liver injury.

## Discussion

In the present study, we utilized the CCl_4_-induced mice liver injury model and the probe drug gigantol to investigate the mechanistic link between the arachidonate metabolic cascade and hepatic inflammation. The inflammatory responses caused by CCl_4_ were accompanied with the activation of MAPK/JNK, cPLA2 and immune cells, which were all reversed by gigantol. The metabonomics analysis suggested that CCl_4_ elevated the concentrations of eicosanoids in the liver, several of which were downregulated by gigantol. In particular, 12-HETE derived from the 12-LOX pathways were most elevated by CCl_4_ and downregulated by gigantol. In short, our study highlighted the role of 12-LOX-12-HETE pathway in the pathogenesis of CCl_4_-induced liver injury which can be extended to other liver diseases. Also, the results suggested that gigantol could be a potential therapeutic for liver inflammation characterized by elevated 12-HETE. Its anti-inflammatory effect at least can be partly attributed to inhibiting JNK/cPLA2/12-LOX pathway.

CCl_4_-induced liver injury model is widely used to investigate the pathological mechanisms of liver injury and evaluate the hepatoprotective effect of potential drug candidates^6^. Oxidative stress (OS) and lipid peroxidation in this model are initially caused by the trichloromethyl and trichloromethyl peroxy radicals, which are the major metabolites of CCl_4_ generated by CYP2E1, CYP2B1 and CYP2B2 in the liver^14^. However, our previous studies suggested that gigantol did not alter the protein expression of Cyp2b9/10 and Cyp2e1 in mouse livers (data not shown). Apart from OS and lipid peroxidation, inflammation is another important mechanism mediating CCl_4_-induced hepatotoxicity. The inflammatory response involves several mediators and cell signaling pathways. During hepatic inflammation, macrophages or neutrophils will be activated or recruited into the liver^15,16^. The activated monocytes will further release ROS, pro-inflammatory cytokines, NO, as well as lipid mediators. In our previous study, the dihydro-stilbene gigantol was found to have potent anti-inflammatory effect both in vitro and in vivo^10^. In this study, we proved that pretreatment of gigantol markedly decreased the M1 polarization of macrophages and inhibited the recruitment of neutrophils. As a result, we selected gigantol as a probe drug to further investigate the involvement of arachidonate metabolic cascade in the process of liver inflammation.

cPLA2 is known to liberate the AA into cytosol with the help of Ca^2+^, which is the rate-limiting step for AA metabolism. So we explored the arachidonate metabolic cascades by starting from the activation of cPLA2. The phosphorylation of cPLA2 was reported to be activated by MAPK/JNK pathway in the H_2_O_2_-induced mouse embryonic stem cells^17^. Since CCl_4_ can activate the MAPK/JNK^7^ and lead to the disruption of intracellular Ca^2+^, we suspected that CCl_4_ could also activate cPLA2 and the subsequent AA metabolism. Just as previous studies^7^, our study showed that JNK was activated at the early stage after CCl_4_ injection, and JNK inhibitor SU3327 could ameliorate the liver injury. When p-JNK level reached the maximum at 2 h post CCl_4_ injection, expression of p-cPLA2 was activated and the activation could last to 16 h. This indicated the activation of cPLA2 was dependent on the MAPK/JNK pathway, and AA metabolites were involved in the liver damage process. JNK activation-induced cPLA2 phosphorylation was inhibited by gigantol at 16 h post CCl_4_ injection, suggesting its inhibitory effect on the AA metabolism.

Subsequently, we investigated the changes in whole AA metabolites in CCl_4_-induced mice liver injury model with the help of UPLC/MS/MS-based targeted metabolomics. The impact of gigantol on these metabolites were also investigated. In mice intoxicated by CCl_4_, the hepatic concentrations of non-enzymatic metabolites (11-HETE), LOX metabolites (5-HETE, 8-HETE, 12-HETE, 15-HETE), CYP450 metabolites (5,6-DHET, 8,9-DHET) and COX metabolites (PGD2, PGH2) were elevated. Gigantol pretreatment resulted in the reduction of 11-HETE, 8-HETE, 12-HETE, 5, 6-DHET and 8, 9-DHET. The inhibition of 11-HETE suggested the downregulation of OS and lipid peroxidation by gigantol. This corresponded with our previous results that gigantol decreased the hepatic malonaldehyde (MDA) and increased superoxide dismutase (SOD) levels^10^. 8-HETE was generated by 8-lipoxygenase (Alox8) and 12-HETE was formed by activated platelet-, and leukocyte-type 12-LOX (Alox12, Alox15). The 5, 6-DHET and 8, 9-DHET were generated from the CYP450 pathways with AA firstly transformed to epoxyeicosatreinoic acids (EETs) by epoxygenase (CYP2C, CYP2J) and then transformed to dihydroxyeicosatrienoic acids (DHETs) by soluble epoxide hydrolase (sEH). HETEs are associated with the pathogenesis of inflammation in numerous diseases, including obesity^18^, diabetes^19,20^, cardiovascular disease^21^ and NASH^22,23^. On the contrary, DHETs and their precursors EETs are proved to have anti-inflammatory effects^24^.

Particularly, the level of 12-HETE was most significantly changed by CCl_4_ and reversed by gigantol. Both platelet-type 12-LOX (Alox12) and leukocyte-type 12-LOX (Alox15) could catalyze the oxygenation of AA at the 12-position to form 12-HETE in mice^25^. The role of 12-LOX has been well demonstrated in the development of diabetes^20,26^. However, its role in liver injury is still vague. Only studies in the mice HIRI model reported that 12-HETE derived from the platelet-type 12-LOX leads to hepatic inflammation. And we also proved that gigantol prevented mice from HIRI, lowering serum ALT and AST, as well as improving liver pathological changes (Supplementary Figure S1). 12-HETE was also found to be elevated in the HBV-induced liver diseases^27^ and NASH^28,29^, but was not investigated thoroughly. To our knowledge, this is the first evidence indicating that 12-LOX-12-HETE is linked to the live toxicity induced by CCl_4_.

At last, the LOX inhibitors were used to verify the role of 12-HETE in the pathogenesis of CCl_4_-induced liver injury. NDGA is a natural product extracted from the *creosote bush* and its inhibitory effects on 5-LOX, 12-LOX and 15-LOX have been well established^30^. It was reported that NDGA could attenuate NASH in a mouse model^31^. In our study, NDGA inhibited the hepatic protein expression of 5-LOX, and platelet-, leukocyte-type 12-LOX activated by CCl_4_ (Supplementary Figure S2). Baicalein could inhibit human platelet-type 12-LOX (Alox12)^12^, which shared 85% sequence homology with mouse platelet-type 12-LOX^26^. ML351 was originally a selective inhibitor for human 15-LOX-1^13^, which shared 73% sequence homology with mouse leukocyte-type 12-LOX (Alox15)^27^. Baicalein has been applied to liver disease models, like HIRI^32^. ML351 has been utilized in some metabolic diseases models, like diabetes^20^, but never been used to prevent liver injury. In this study, NDGA, baicalein and ML351 attenuated the liver injury to the same extents as gigantol. The results verified the LOXs, especially 12-LOX as potential therapeutic target for acute liver injury. In the future, further investigations should be conducted to figure out whether gigantol can directly inhibit the activity of 12-LOX (platelet, leukocyte-type).

**Figure 8:**
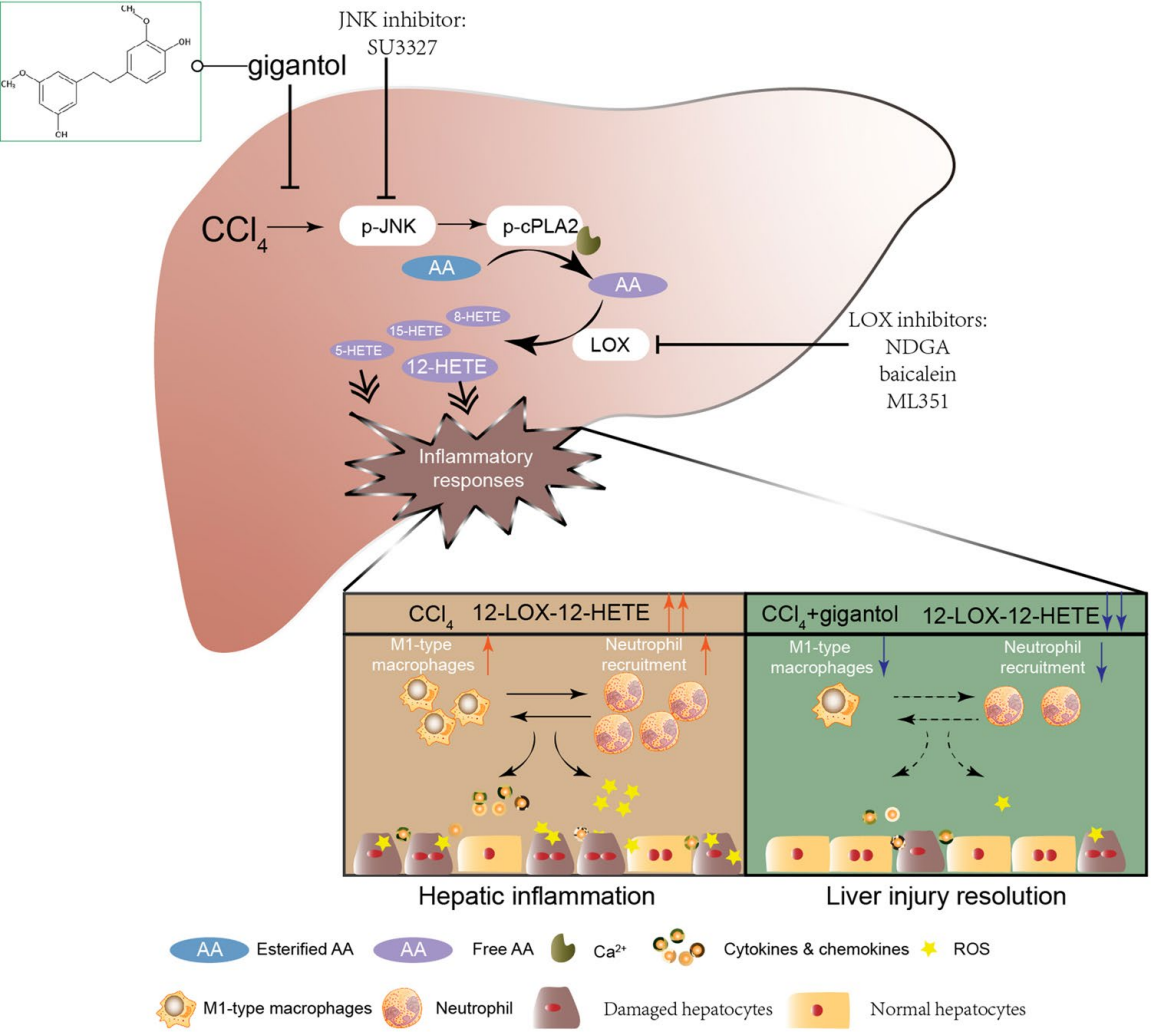
Schematic diagram of arachidonate metabolic cascade in liver inflammation and the intervention strategies. CCl_4_ activates AA metabolism and leads to increased M1-type macrophages and neutrophils, as well as elevated cytokines, chemokines and ROS levels in the liver. Oral administration of gigantol alleviates liver inflammation partly through inhibiting the JNK/cPLA2/12-LOX pathway. The image was created by using Adobe Illustrator CC 2019 (https://www.adobe.com/products/illustrator.html).

In summary, we elucidated a comprehensive profile of AA metabolites in the acute liver injury model caused by CCl_4_ for the first time. 12-LOX-12-HETE pathway was highlighted in the pathogenesis of hepatotoxicity caused by CCl_4_. We further validated the anti-inflammatory effect of gigantol from the respect of immune cells and AA metabolism. Our results suggest that oral administration of gigantol alleviates the activation of immune cells and AA metabolism in CCl_4_-induced mouse livers. And its hepatoprotective mechanism can be partly attributed to the inhibition of JNK/cPLA2/12-LOX pathway (Fig. 8).

## Methods

### Chemicals and biochemical reagents

Carboxymethylcellulose sodium salt (CMC-Na), carbon tetrachloride (CCl_4_), olive oil, Tween 80, formaldehyde, ethanol, acetic acid, isopropanol and NH4OAC were purchased from Sinopharm Group Co., Ltd. (Shanghai, China). Acetonitrile and methanol (high-performance liquid chromatography grade) were purchased from Thermo Fisher Scientific (Madison, WI, USA). Soluto HS15, β-cyclodextrin were obtained from Meilun Biotechnology (Dalian, LiaoNing, China). Nordihydroguaiaretic acid (NDGA) and baicalein were purchased from Santa Cruz Biotechnology (Santa Cruz, CA, USA). ML351 was brought from Cayman Chemical (Ann Arbor, MI, USA). Gigantol was synthesized according to a previous report^33^ and kindly granted by Professor Yang Ye (Shanghai Institutes of Materia Medica, SIMM).

Rabbit monoclonal antibodies against GAPDH (#2118), phospho-SAPK/JNK (Thr183/Tyr185) (#4668) and rabbit polyclonal antibody against SAPK/JNK (#9252) were obtained from Cell Signaling Technology (Danvers, MA, USA). Mouse monoclonal antibody against platelet-type 12-LOX (#sc-365194) and leukocyte-type 12-LOX (#sc-133085) were obtained from Santa Cruz Biotechnology (Santa Cruz, CA, USA). Rabbit polyclonal antibodies against cPLA2 (#ab58375) and phospho-cPLA2 (Ser505) (#ab53105) were purchased from Abcam (Cambridge, MA, USA). HRP-conjugated goat anti-mouse (#Abs2001) and goat anti-rabbit IgG (#Abs2002) were purchased from Absin Bioscience (Shanghai, China). Antibodies against PE-Cy7-F4/80 (#25-4801-82) and PE-Ly-6G (#12-9668-80) were brought from eBiosciences (San Diego, CA, USA). Antibody against APC-CD86 (#558703) was brought from BD Pharmingen (San Diego, CA, USA). Antibodies against FITC-CD206 (#141703) and FITC-CD11b (#101205) were from Biolegend (San Diego, CA, USA).

### Animals

Male ICR mice and C57 mice were bought from the Shanghai Slac Laboratory Animal Co. Ltd. The mice were kept in air conditioned animal quarters with food and water ad libitum. The environment was maintained at a controlled temperature (23 ± 1.5 °C) and humidity (70% ± 20%). All the animal experiments in this study were approved by Institutional Animal Care and Use Committee of SIMM, Chinese Academy of Sciences (CAS) (Approval no: 2018-06-PGY-25) and performed in accordance with the approved ethical guidelines.

### CCl_4_-induced liver injury model

After a single subcutaneous injection of CCl_4_ (10 mL kg^−1^ as 0.5% in olive oil), the ICR mice (24–26 g) were sacrificed at the indicated time points. The JNK inhibitor SU3327 (10 mg kg^−1^, a single *ip* injection) was prepared with 10% Solutol HS15 in PBS according to previous report ^7^. Gigantol (40 mg kg^−1^, dissolved in 0.5% Tween 80 aqueous solution) and the pan-LOX inhibitor NDGA (40 mg kg^−1^, dissolved in 10% β-cyclodextrin in saline) were intragastrically administrated for 7 days. Baicalein (40 mg kg^−1^, suspended in 0.5% CMC-Na aqueous solution) was administrated for 7 days in a way of intraperitoneal injection. The leucocyte-type 12-LOX inhibitor ML351 (40 mg kg^−1^) was prepared in 3% ethanol/17% cremophor EL/PBS^20^ and administrated for 3 times at intervals of 8 h. Generally, the dose of gigantol and other inhibitors were selected based on their protective effects on liver injury and inhibitory effects on relevant signaling pathways. All the doses selected were well tolerated by ICR mice. The dose of gigantol was determined according to our previous study^10^. The dose of NGDA was equal to previous study conducted in NASH model^31^. The doses selected for baicalein and ML351 were comparablely to that reported in previous studies and supposed to downregulate the 12-HETEs levels in plasma^20,34,35^. The last administration of the above compounds or vehicle was conducted 1 h before CCl_4_ injection. Blood and liver samples were collected at the indicated time post CCl_4_ administration. The blood will be centrifuged at 8000 rpm for 10 min to get serum samples. Serum levels of ALT and AST were measured with the commercial kits obtained from Nanjing Jiancheng Institute of Biotechnology (Nanjing, Jiangsu, China). One part of the liver samples was quickly frozen with liquid nitrogen and used for further biochemical analysis. The other part of the livers was fixed with formalin. To evaluate the histological changes, liver tissues were embedded in paraffin and sliced into 3-μm thick sections. The sections were stained with hematoxylin and eosin (H&E) and images were scanned by Hamamatsu Photonics NanoZoomer 2.0 HT digital slide scanner (Hamamatsu, Shizuoka, Japan).

### Hepatic ischemia reperfusion injury (HIRI) model

C57 mice (22–25 g) were randomly divided into three groups: the sham-operated group; I/R model group; and gigantol pretreatment group. The mice were intravenously injected with gigantol (40 mg kg^−1^) 3 times every 12 h. The mice in the sham and I/R model groups were administered with solvent (0.5% Tween 80). Surgery was performed 30 min after the last administration. The mice were anaesthetized by pentobarbital sodium (50 mg kg^−1^, *ip*) and then subjected to 70% warm hepatic I/R surgery following the procedure described in previous studies^36^. Briefly, the mice were mildly laparotomized to expose the liver, and the Glisson system containing the hepatic portal vein, hepatic artery and bile duct was separated. The Glisson system was occluded by an atraumatic microvascular clamp (Fine Science Tools, North Vancouver, BC, Canada), leading to ischemia of the left and median lobes. After 1 h of ischemia, the clamp was removed for another 6 h of reperfusion. The mice in the sham groups underwent a similar operation except vasculature occlusion. Blood samples and liver tissues were collected after 6 h of reperfusion.

### SDS-PAGE and immunoblot analysis

The protein samples were extracted from the frozen livers according to previous protocols^37^. The samples dissolved in the Yeasen Biotech sodium dodecyl sulphate (SDS)-loading buffer (Shanghai, China) were electrophoresed and transferred to Merck Millipore polyvinylidene difluoride (PVDF) membranes (Billerica, MA, USA) and then subjected to immunoblot analysis with the antibodies against p-JNK, JNK, p-cPLA2, cPLA2, platelet-type 12-LOX and leukocyte-type 12-LOX. The images were captured by the CLINX ChemiScope 3300 mini system (Shanghai, China). The relative protein abundance in each group was quantified by National Institute of Health Image J software (Silver Springs, MD, USA).

### Real-time PCR analysis

The frozen livers were used to extract RNA samples with the Life Technology TRIzol reagent (CA, USA) and the Sangon Biotech EZ-10 Spin column & Collection Tubes (Shanghai, China). cDNA was obtained using Takara PrimeScript RT Master Mix (Tokyo, Japan) and amplified by the Yeasen Biotech Hieff qPCR SYBR Green Master Mix (Low Rox Plus)(Shanghai, China). Thermal cycling was performed in the Applied Biosystems 7500 Fast real-time PCR System (FosterCity, California, USA). The relative mRNA expression was quantified using the 2^−ΔΔCT^ method with GAPDH as the internal control. The primers sequences were listed in Supplementary Table S2.

### Flow cytometric analysis

The fresh liver tissues were grinded to prepare the cell suspension. The M1-type macrophages were double-labeled by F4/80 and CD86. The M2-type macrophages were double-labeled by F4/80 and CD206. The infiltrated neutrophils were double-labeled by CD11b and Ly6G. All the staining processes were conducted according to the manufacturer’s protocol, and then the stained cells were subjected to Fluorescence-Activated Cell Sorting (FACS) assay with the BD Calibur flow cytometer (Franklin Lakes, NJ, USA). The off-line analysis was performed using TreeStar FlowJo 7.6 software (Ashland, OR, USA).

### Metabonomics

The frozen livers from mice in the vehicle, gigantol alone, CCl_4_, CCl_4_ plus gigantol groups were subjected into the AA metabonomics analysis. A section of liver tissues was cut and homogenated with ice water (w/v = 1:5) and then sonicated for 15 min. A mixture of methanol: acetonitrile (1:1, v/v) was used to extract the AA and AA metabolites. After thorough vortex, the sample was centrifuged at 14,000 rpm, 4 °C for 10 min, and then 30 μL of the supernatant was mixed with 30 μL of water for LC/MS analysis. Samples were separated by the Waters CQUITY H–Class UPLC System (Milford, MA, USA) and analyzed by AB Sciex triple quadrupole 6500 mass spectrometer (FosterCity, CA, USA). The Waters ACQUITY UPLC Tag ultra C18 (1.7 μm, 2.1 mm × 100 mm) was used for the analysis. Gradient elution was used with a mobile phase composed of solvent A (water containing 0.1% acetic acid and 1 mM NH_4_OA_C_) and solvent B (acetonitrile: isopropanol (1:1, v/v)).

### Statistical analysis

All the data were analyzed by GraphPad Prism 7.0 software (La Jolla, CA, USA) and shown as Mean ± SD unless other statement. One-way analysis of variance (ANOVA) was used to compare the differences between more than three groups. Statistical analyses between two groups were performed using the Student's *t* test. *P* < 0.05 represents statistical difference.

## Supplementary information

Supplementary Information 1.
