# Supplementary material for: Gigantol ameliorates CCl4-induced liver injury via preventing activation of JNK/cPLA2/12-LOX inflammatory pathway
Source: Sci Rep. 2020 Dec 17;10:22265. doi: 10.1038/s41598-020-79400-0 (PMC7746690; doi:10.1038/s41598-020-79400-0)
Supplement: Supplementary file 1 — Supplementary Information 1. [file 41598_2020_79400_MOESM1_ESM.pdf]

Gigantol ameliorates CCl_4-induced liver injury via preventing activation of JNK/cPLA2/12-LOX inflammatory pathway

Yaru Xue[1,2], Qiangqiang Deng[1], Qingli Zhang[3], Zhenghua Ma[4,5,6], Binfan Chen[1,2], Xiaolu Yu[1,2], Huige Peng[1], Sheng Yao[1,4,5], Jia Liu[3], Yang Ye [1,4,5,6*], Guoyu Pan[1,2*]

[1]Shanghai Institute of Materia Medica, Chinese Academy of Sciences, Shanghai 201203, China. [2]University of Chinese Academy of Sciences, Beijing 100049, China. [3]Institutional Technology Service Center, Shanghai Institute of Materia Medica, Chinese Academy of Sciences, Shanghai, 201203, China. [4]State Key Laboratory of Drug Research and Natural Products Chemistry Department Shanghai Institute of Materia Medica, Chinese Academy of Sciences, Shanghai 201203, China. [5]SIMM-CUHK Joint Research Laboratory for Promoting Globalization of Traditional Chinese Medicines, Shanghai 201203, China. [6]School of Life Science and Technology, Shanghai Tech University, Shanghai 201203, China. [*]E-mail: gypan@simm.ac.cn; yye@simm.ac.cn.

Supplementary data

Supplementary Table S1. The fold changes of AA and its metabolites concentration between the vehicle group and the CCl_4 group or the CCl_4 group and the (CCl_4+gigantol) group. $^{###}P<0.001$, $^{##}P<0.01$, $^{#}P<0.05$ the CCl_4 group *vs.* the vehicle group; $^{**}P<0.01$, $^{*}P<0.05$ the (CCl_4+gigantol) group *vs.* the CCl_4 group; +∝ means positive infinity; n=6 mice for each group.

AA and AA metabolites	CCl_4/vehicle	CCl_4/(CCl_4+gigantol)
AA	$1.33^{#}$	1.08
9-HETE	2.26	1.77
11-HETE	$2.92^{##}$	1.70*
5-HETE	$4.08^{###}$	1.22
8-HETE	$+\propto^{###}$	2.11**
12-HETE	$6.67^{#}$	2.85*
15-HETE	$2.58^{##}$	1.68
LTC_4	0.61	1.24
20-HETE	1.12	1.68*
5, 6-DHET	$4.45^{###}$	1.48**
8, 9-DHET	$2.06^{###}$	1.34**
PGD2	$3.01^{#}$	1.17
PGF2	1.06	0.95
PGH2	$4.72^{#}$	1.11
6-keto-PGF1α	3.58	1.23
TXB2	3.15	1.05
PGE2	1.86	0.98

Supplementary Table S2. The primers sequences for real-time PCR

Gene name	Direction	Sequence
GAPDH	Forward	AAGTGCATCATCGTTGTTCATACA
	Reverse	GAGGATACCACTCCCAACAGACC
PLA2	Forward	GCAGGCAGAGCGATATGATG
	Reverse	CAGCTCCGTCTCGATCTTCT
Alox5	Forward	AACGATCACCCACCTTCTGC
	Reverse	TCGCAGATAAGCTGTTCCCG
Alox12	Forward	TCCCTCAACCTAGTGCGTTTG
	Reverse	GTTGCAGCTCCAGTTTCGC
Alox15	Forward	TGGATGGGATCAAGGCCAAT
	Reverse	ATCCAAGGGCGTGAAAATCG
COX2	Forward	CAGGTCATTGGTGGAGAGGTGTATC
	Reverse	CCAGGAGGATGGAGTTGTTGTAGAG

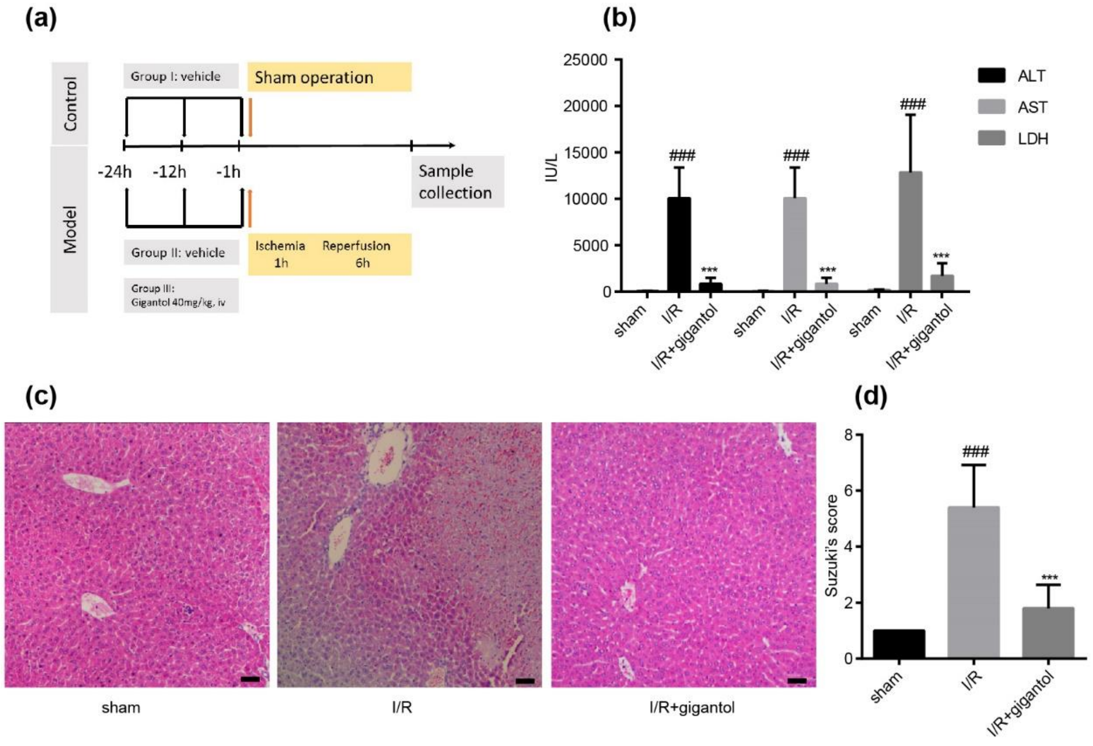

Figure S1. Protective effect of gigantol on an HIRI model. (a) An overview of the experimental scheme used for the HIRI. (b) Serum ALT, AST and LDH levels. (c) Representative histological sections of liver tissues stained with H & E. Symbols: black arrow- centrilobular necrosis; rectangle-tumefaction and steatosis; solid triangle-inflammatory infiltration; circle-congestion. CV-centrilobular veins; PV-portal veins. Scale bar = 50 μm. (d) The quantitative results of H & E staining, as determined by Suzuki scores. Representative results are shown as the mean ± SD, n=6 mice in each group. ###P<0.001 *vs.* the sham group; ***P<0.05 *vs.* the I/R group.

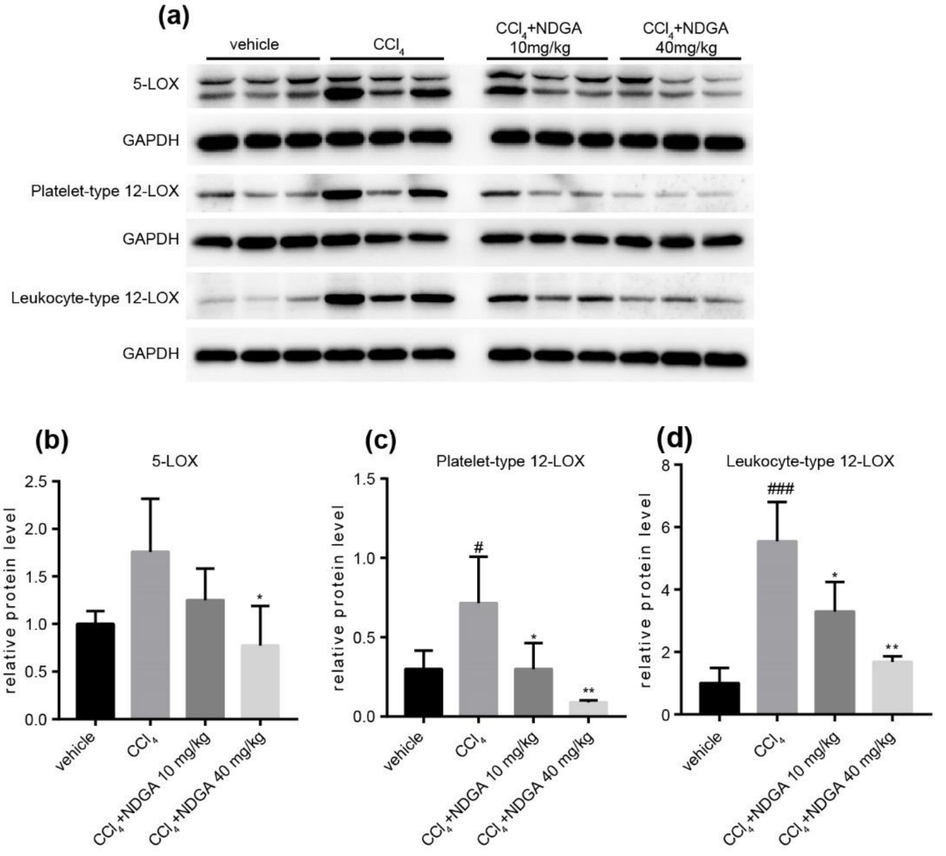

Figure S2. Inhibitory effect of NDGA on the protein expression of lipoxygenases. NDGA (10, 40 mg/kg, dissolved in 10% β-cyclodextrin in saline) was intragastrically administrated for 7 days before CCl_4 induction. (a) The representative western blot bands of 5-LOX, platelet- and leukocyte-type 12-LOX in mouse livers , n=3 experiments. (b-d) The quantitative results are represented as the mean ± SD, n=6 mice in each group. ###P<0.001, #P<0.05 *vs.* the vehicle group; **P<0.01, *P<0.05 *vs.* the CCl_4 group.

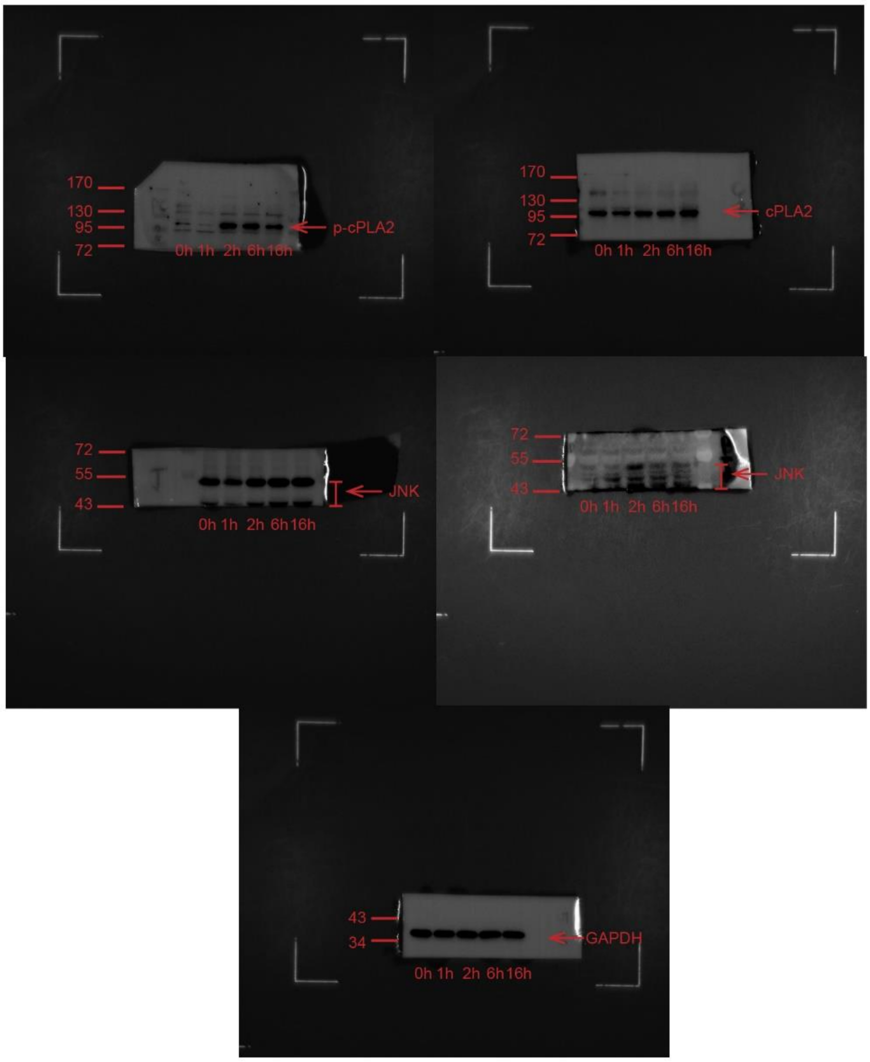

Figure S3. Uncropped images of Figure 1B

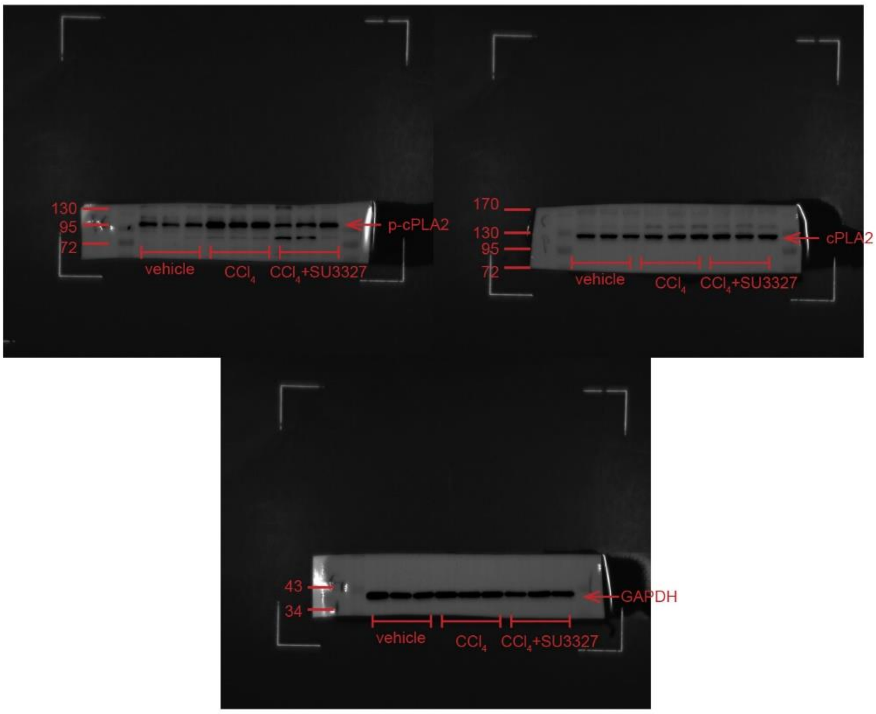

Figure S4. Uncropped images of Figure 1D

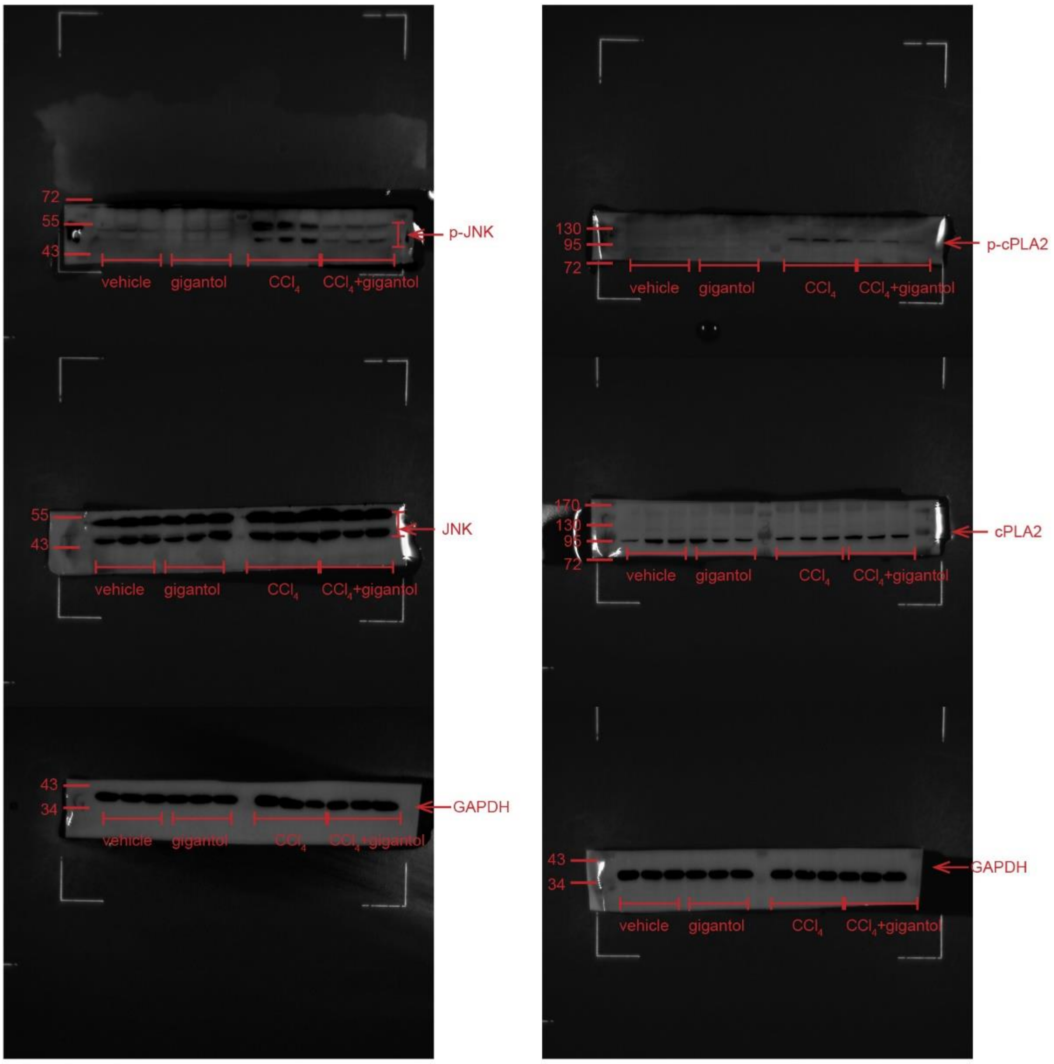

Figure S5. Uncropped images of Figure 3A

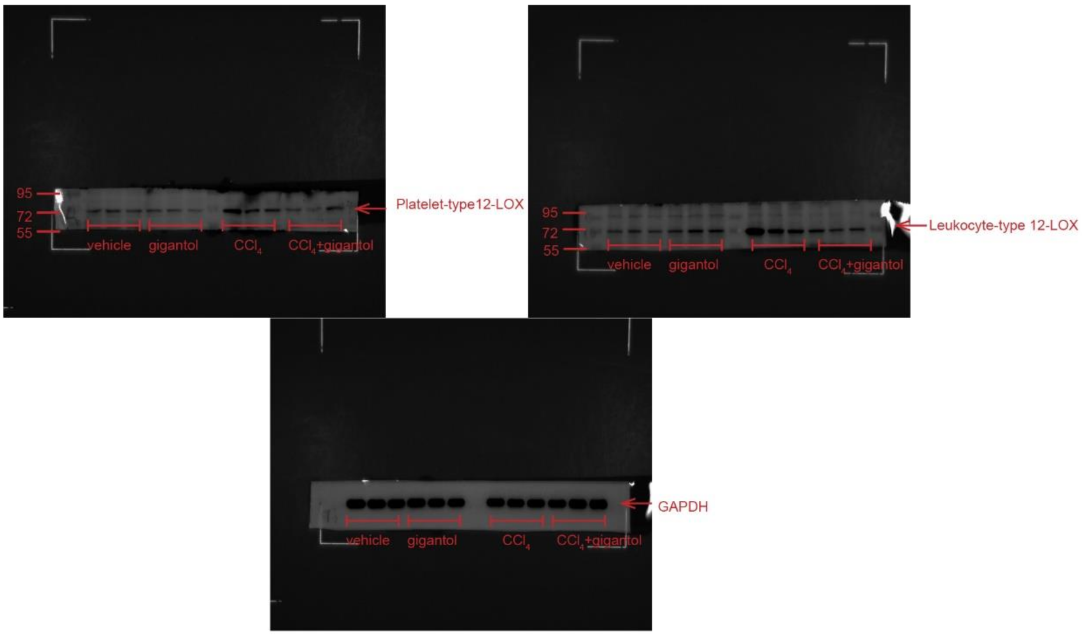

Figure S6. Uncropped images of Figure 5B

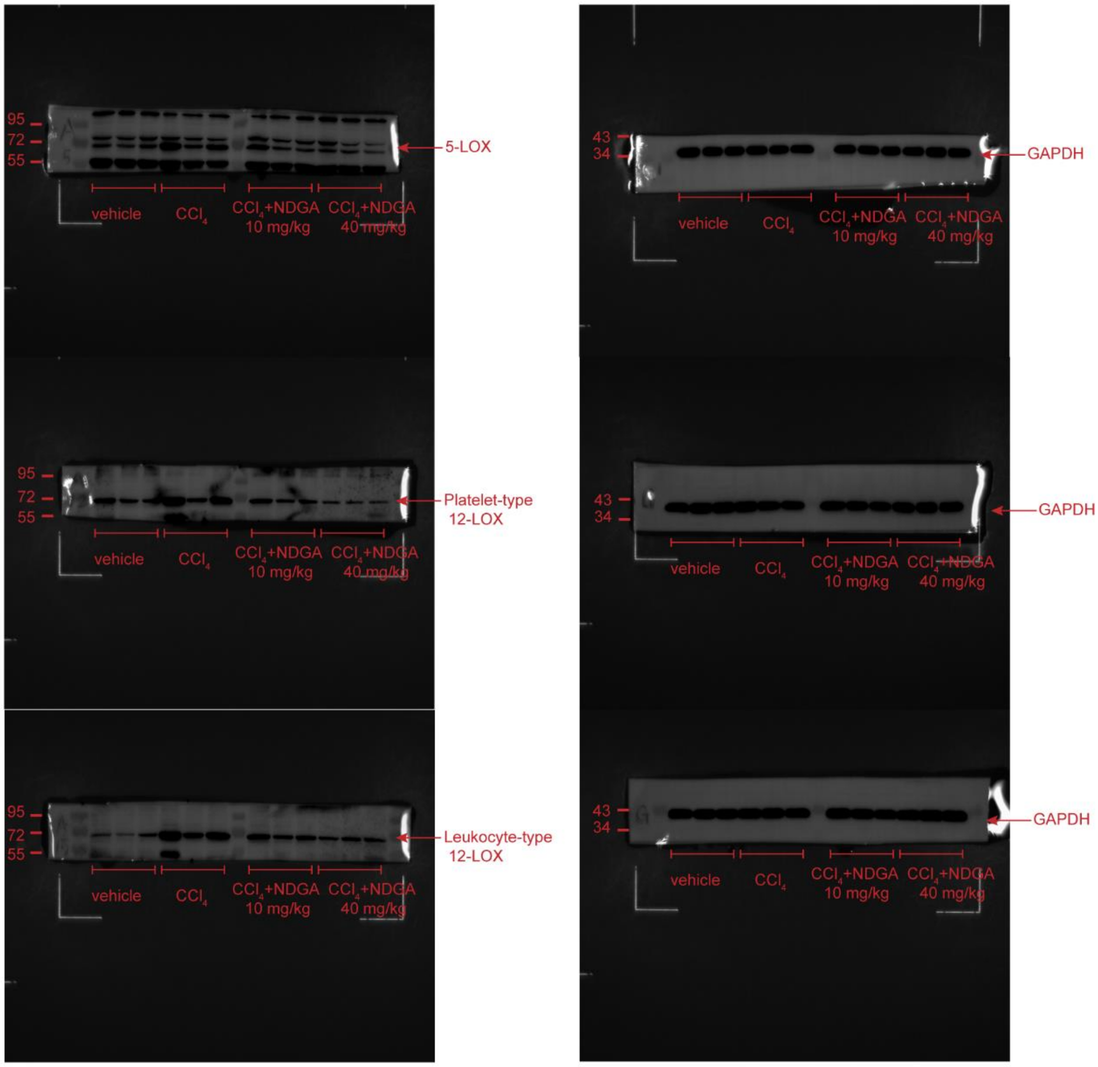

Figure S7. Uncropped images of Figure S2
